# In Vivo Anatomical Variations in the Lateral Femoral Cutaneous Nerve in Children

**DOI:** 10.3390/children12040521

**Published:** 2025-04-17

**Authors:** Lise Langeland Larsen, Line Kjeldgaard Pedersen, Ole Rahbek, Bjarne Møller-Madsen

**Affiliations:** 1 Department of Children’s Orthopaedics, Aarhus University Hospital (AUH), 8200 Aarhus, Denmark; linepede@rm.dk (L.K.P.); bj.moma@aarhus.rm.dk (B.M.-M.); 2 Department of Orthopedics, Randers Regional Hospital, 8930 Randers, Denmark; 3 Department of Children’s Orthopaedics, Aalborg University Hospital (AAUH), 9000 Aalborg, Denmark; o.rahbek@rn.dk

**Keywords:** surgery, pediatric, children, nerve, anatomy, lateral femoral cutaneous nerve

## Abstract

Background: The anatomic pathways of the lateral femoral cutaneous nerve (LFCN) have primarily been reported in adult in vitro populations with limited branching patterns. Children with hip disorders may require surgical treatment with an anterior approach, and the LFCN is a structure at risk. The aim of our study was to photographically verify the initial six-centimeter pathway of the LFCN in children measured from its appearance at the anterior superior iliac spine (ASIS). Method: A total of 31 children underwent pelvic osteotomy, including three bilateral. The nerve was identified and isolated in the subcutaneous layer. Standardized photographs were obtained. Our outcome parameters were type of pelvic exit, branching pattern, distance from the pelvic exit to the ASIS, and nerve thickness and appearance, categorized as straight or curved. Results: 91.3% of nerves passed medially to the ASIS. A total of 74% of the nerves showed a splitting branching pattern, and 9% had a branching pattern of more than four. The mean distance from pelvic exit to the ASIS was 17 mm, and the mean nerve thickness was 2.7 mm. Conclusions: In contrast to adult anatomy, our study shows that the LFCN has two or more branches in 74% of patients. Thus, based on our observations, surgeons should carefully dissect the subcutaneous tissue around the LCFN due to the numerous anatomical variations to avoid iatrogenic damage to the nerve.

## 1. Introduction

The lateral femoral cutaneous nerve (LFCN) is a purely sensory nerve that normally derives from the posterior division of L2 and L3 and innervates the anterolateral aspect of the thigh. The LFCN is at risk during surgical procedures, e.g., the modified Smith-Petersen approach to the hip. Iatrogenic injury to the nerve is the most common cause of meralgia paresthetica (MP) [1]. Incidence of MP in adults undergoing hip surgery ranges from 30 to 74% [2,3]. MP expresses itself from a tingling or burning sensation in the anterolateral aspect of the thigh to a disabling chronic pain syndrome [1].

MP among children is considered rare but may be more common with pain as the main symptom [4]. Electromyographic examination of the nerve has shown a high incidence of injury to the nerve after an anterior approach to the hip [5]. Several anatomic pathways of the LFCN in adult cadavers have been described [6,7,8,9,10]. However, the pediatric course of the LCFN has only been described from normalized data from adults and in one small in vivo study [11,12].

Therefore, our aim in this study was to visualize the most proximal anatomy of the LFCN from its appearance at the anterior superior iliac spine (ASIS) by photography during in vivo surgery.

## 2. Materials and Methods

**Figure 1:**
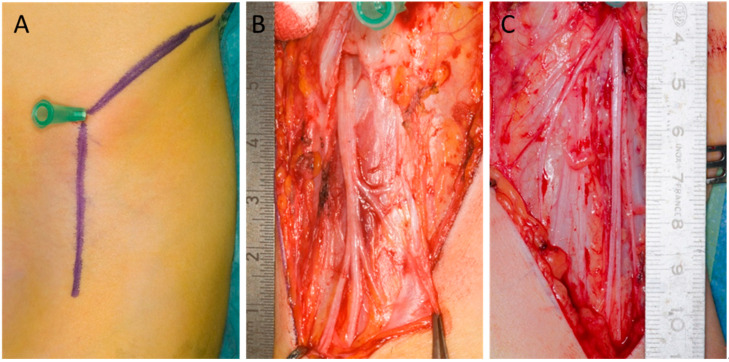
Pelvic exit (simplified type A and B). (**A**) A modified Smith-Petersen approach was used in all surgeries. The needle marks the ASIS (anterior superior iliac spine). (**B**) Left side LFCN (lateral femoral cutaneous nerve) (same patient as (**A**)). The pelvic exit is medial to the ASIS, according to a simplified type A classification. The LFCN exits 23 mm from the SIAS. Thickness: 2.5 mm. Minimum of six branches with straight appearances. (**C**) Right side LFCN. The pelvic exit is directly cranial to the ASIS according to a simplified type B. The LFCN exits 3 mm from the ASIS. Thickness 5 mm Minimum of six branches with straight appearances.

This was a single-institution clinical case-series study. Thirty-one children (15 boys, 16 girls) 4–14 years old, who underwent pelvic osteotomy (three bilateral), were included in this study. Twenty-five children were Caucasian, five were Asian, and one was South American. All skin incisions were modified Smith-Petersen approaches. The LFCN was identified in the subcutaneous tissue under the fibrous membrane, which was barely visible. The nerve was dissected free of adjacent soft tissue. ASIS was marked by a syringe, and a ruler was placed alongside the proximal six centimeters of the nerve. Standardized photographs was obtained (Figure 1). Statistical analysis included descriptive statistics with numbers and percentages.

The outcome parameters were (a) pelvic exit categorized according to the seven types of exits published by Tomaszewski et al. (2016) [8], (b) branching pattern, (c) the distance from the pelvic exit to the ASIS, (d) LFCN thickness, and (e) LFCN appearance categorized as straight or curved. A simplified classification of pelvic exit was applied as complete dissection was impossible due to the in vivo nature of the study (Table 1). Type A: pelvic exit medial to the ASIS (Tomaszewski type 1, 2, 3, and 7); type B: pelvic exit directly over the ASIS (Tomaszewski type 4 and 6); and type C: pelvic exit lateral to the ASIS (Tomaszewski type 5) (Figure 1). The anatomical dissection of the LCFN in the present study was considered to be without excess risk for the patients in the study. The study was not considered a health research study by the Central Denmark Region Committee on Health Research Ethics; hence, the study was conducted without approval.

## 3. Results

### 3.1. Pelvic Exit (Figure 1)

In simplified classification, 93.1% had an exit that was medial to the SIAS (type A), 4.3% cranial to the SIAS (type B), and 2.6% lateral to the SIAS (type C). A total of 91% left the pelvis medially to the SIAS and profoundly to the sartorius muscle (type 1), one nerve left the pelvis medially to the SIAS but through the inguinal ligament (type 2), one nerve left through the ASIS (type 6), and one nerve left laterally to the ASIS (type 5).

### 3.2. Branching Pattern (Figure 2)

A total of 9% had a branching pattern of more than four, 6% showed quadrification, 20% showed trifurcation, 38% showed bifurcation, and 26% showed no branching. Of these, 35% can be defined as fan-types, which in the literature are defined by multiple branches.

**Figure 2:**
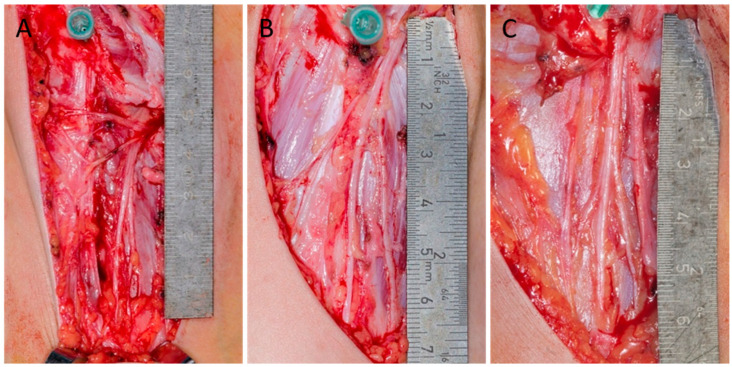
Examples of branching patterns and LFCN (lateral femoral cutaneous nerve) appearance. The needle marks the ASIS (anterior superior iliac spine), and the ruler is placed medially to the ASIS. (**A**) A right LFCN simplified type A classification with curved appearance and at least quadrification. (**B**) A right LFCN simplified type A classification with a slightly curved appearance and at least trifurcation. (**C**) A right LFCN simplified type A classification with a linear appearance and at least trifurcation.

### 3.3. Distance from Pelvic Exit and Nerve Appearance

The mean distance from the pelvic exit to the ASIS was 17 mm, and the mean thickness of the LFCN was 2.7 mm. Nine nerves (26%) were curved, and 25 nerves (74%) were straight.

Damage to the LFCN was not systematically recorded; however, no damage was clinically apparent in any of the patients to the LFCN during and/or after surgery.

## 4. Discussion

The present in vivo study visualizes the pathways of the LFCN at the proximal thigh in 34 children undergoing a modified Smith-Petersen skin incision.

The vast majority of previous publications focused on the anatomy of the LFCN are based on observations from adult cadaver studies. In contrast to most anatomical textbooks, we observed a large variability of the pelvic exit location and also the propagation of the number and thickness of nerve branches distally on the anterolateral thigh. Unfortunately, anatomical textbooks give insufficient detailed information about the distribution pattern of the LFCN in the proximal region of the thigh. In the worst case, this may lead to unwanted surgical injury to the nerve and potentially MP. Pelvic exit medial to the sartorius muscle was predominantly high (91%), but in accordance with Tomaszewski et al. [7]. The number of branches varied considerably in accordance with previously published data [1,9,13]. The fan type may be challenging operatively, as the approach to the hip most conveniently goes between the tensor fascia latae and sartorius muscle. However, the clinical implications of the branching pattern are unknown. Whether a high number of branches is a risk factor or a protective factor of MP is yet to be discovered. Rudin et al. reported the individual distribution pattern of the nerve, the skin incision, and the tissue dissection technique to increase the risk to the LFCN and concluded that an injury to the nerve cannot be avoided with an anterior approach to a hip with a fan-type LFCN [13]. This is in concordance with Ozaki et al., who, by postsurgical questionaries, described nerve damage in nine of ten patients with Fan-type LFCN in contrast to ten of 25 with a non-Fan type using an anterior approach to the hip [14]. Kiyama et al. report that neural ischemia due to medial nerve retraction in the modified Smith-Petersen approach in adults causes a reduced blood flow in the area of the LFCN, with 74% of patients experiencing sensory disturbance at two weeks postoperative and 11% at one-year follow-up [15]. A high branching pattern could cause lower traction on the nerve branches during surgery and hence protect the nerve from ischemia and subsequent damage. In order to reduce the risk of nerve injury, further studies are needed, e.g., ultrasound application. Ultrasound is a validated and applicable method to examine the branching type of the LFCN [14,16,17,18]. Concerning the distance from the pelvic exit to the ASIS, our observation is in accordance with previously published data [1,11,12]. The present study has a few limitations. First, there was a limited number of included patients. Second, a complete postoperative clinical evaluation of sensory function was not possible due to the majority of participants having varying degrees of cognitive impairment due to cerebral palsy. Finally, we assumed the general anatomy did not vary among included patients; however, this cannot be ruled out due to potential abnormal rotational profiles.

## 5. Conclusions

In a pediatric population, we found that the LCFN exhibited a splitting branching pattern in 75% of patients compared to only 20% in adults. The number of branches was more than 4 in 9% of patients. Thus, surgeons should carefully dissect the subcutaneous tissue around the LCFN due to the numerous anatomical variations.

## Figures and Tables

**Table 1:** Type of pelvic exit, branching pattern, and simplified type of pelvic exit. Results from the present study in comparison to observations given in the meta-analysis by Tomaszewski et al. [8]. IL (inguinal ligament), ASIS (anterior superior iliac spine).

	Present Study (Pediatric Population)	Study by Tomaszewski et al. (Adult Population)
Type of pelvic exit	*n* = 34	*n* = 1473
1. Medial to m. Sartorius: % (95% CI)	91	86.8 (71.7–90.0)
2. Through IL: % (95% CI)	3	3.7 (0.2–9.4)
3. Proximal to IL: % (95% CI)	0	0.9 (0.0–3.6)
4. Cranial to ASIS: % (95% CI)	0	1.9 (0.0–5.6)
5. Lateral to ASIS: % (95% CI)	3	2.6 (0.0–6.7)
6. Through the ASIS: % (95% CI)	3	2.4 (0.0–6.4)
7. Through sartorius: % (95% CI)	0	1.7 (0.0–5.2)
Branching pattern	*n* = 34	*n* = 993
No splitting: % (95% CI)	26	79.1 (58.7–85.0)
Bifurcation within pelvis: % (95% CI)	3	11.8 (3.1–21.9)
Bifurcation in IL area: % (95% CI)	35	3.3 (0.0–9.1)
Trifurcation: % (95% CI)	20	4.8 (0.0–11.4)
Quadrification: % (95% CI)	6	1.0 (0.0–4.8)
More than four branches: % (95% CI)	9	n.a.
Simplified type of pelvic exit	*n* = 34	*n* = 1473
Type A (Medial to ASIS)	94	93.1
Type B (Over ASIS)	3	4.3
Type C (Lateral to ASIS)	3	2.6

n.a.: No documented cases of more than four branches.

## Data Availability

The original contributions presented in the study are included in the article, further inquiries can be directed to the corresponding author.
